# Manganese – a scoping review for Nordic Nutrition Recommendations 2023

**DOI:** 10.29219/fnr.v68.10367

**Published:** 2024-01-16

**Authors:** Maria Kippler, Agneta Oskarsson

**Affiliations:** 1 Institute of Environmental Medicine, Karolinska Institutet, Stockholm, Sweden; 2 Department of Biomedical Sciences and Veterinary Public Health, Swedish University of Agricultural Sciences, Uppsala, Sweden

**Keywords:** manganese, minerals, trace elements, nutrition recommendations

## Abstract

Manganese is an essential trace element that is required for multiple enzymes in the human body. The general population is mainly exposed to manganese via food intake, in particular plant foods. In areas with elevated concentrations of manganese in groundwater, drinking water can also be an important source of exposure. The gastrointestinal absorption of manganese is below 10%, and it appears to be influenced by the amount of manganese in the diet and by the nutritional status of the individual, especially the iron status. In blood, most of the manganese is found in the cellular fractions. Manganese is primarily eliminated via the bile followed by excretion via faeces. To date, no specific biomarkers of manganese intake have been identified. The dietary intake of manganese in the Nordic countries has been reported to be within the range that has been reported for other European countries (2–6 mg/day). Since manganese is found in nutritionally adequate amounts in food, deficiency is not of public health concern. On the other hand, there is emerging epidemiological evidence that various suggested manganese biomarkers may be negatively associated with children’s neurodevelopment. However, the limited number of prospective studies, the lack of appropriate exposure biomarkers, and validated neurodevelopmental outcomes render data uncertain and inconclusive. In 2013, the European Food Safety Authority considered the evidence to be insufficient to derive an average requirement or a population reference intake, and instead an adequate intake for adults was set at 3.0 mg/day.

## Popular scientific summary

- Manganese is an essential trace element for humans.
- In humans, manganese is involved in the synthesis and activation of multiple enzymes, and it is required for metabolism.
- The main dietary sources are cereals, vegetables, and fruits, but drinking water can contribute in some areas.
- Deficiency is not of public health concern.
- No qualified biomarker for manganese status has been established.

Manganese is a naturally occurring element. It is the twelfth most abundant element in the Earth’s crust. Manganese is widely distributed in the environment both through natural processes and anthropogenic activities (e.g. production of steel, batteries, chemicals, glass and fireworks, and the use of fertilizers) (1). Therefore, manganese can occur in airborne and deposited particles, water, and soil, and it is actively absorbed by plants.

Manganese is an essential trace element for mammals. It can exist in several oxidation states ranging from −3 to +7, but in mammals the dominating forms are Mn^2+^ and Mn^3+^. Mn^2+^ is the most stable form, whereas Mn^3+^ is an oxidant that is either reduced to Mn^2+^ or forms complexes with proteins. In the human body, manganese is involved in the synthesis and activation of multiple enzymes (e.g. oxidoreductases, transferases, hydrolases, lyases, isomerases, and ligases), and it is a required cofactor in a variety of metalloenzymes, including arginase, pyruvate carboxylase, glutamine synthetase, and manganese superoxide dismutase (MnSOD) (2). Manganese is found in all tissues, and it is required for normal amino acid, lipid, protein, and carbohydrate metabolism as well as for maintenance of mitochondria through scavenging of reactive oxygen species. It is also involved in reproduction, bone formation, immune function, regulation of blood sugars and cellular energy, digestion, and in blood clotting together with vitamin K (2).

The general population is mainly exposed to manganese via intake of food and manganese-containing supplements. Drinking water is another important source of exposure in areas with elevated concentrations of manganese in groundwater. Certain individuals may be exposed through parenteral nutrition or through residing in a contaminated area or via direct occupational exposure leading to inhalation of airborne particles or fumes (1). It is well established that long-term elevated occupational exposure to manganese may be associated with neurotoxicity, which manifests as cognitive and movement disorder similar to Parkinson disease (3). There is also emerging evidence suggesting that consumption of drinking water with elevated manganese concentrations may be associated with developmental neurotoxicity (4, 5). As manganese is ubiquitously found in high enough concentrations in food, deficiency is not of public health concern.

The aim of this scoping review is to describe the totality of evidence for the role of manganese for health-related outcomes as a basis for setting a dietary reference value (DRV) for the Nordic Nutrition Recommendations (NNR) 2023 (Box 1).

*Box 1.* Background papers for Nordic Nutrition Recommendations 2023

- This paper is one of many scoping reviews commissioned as part of the Nordic Nutrition Recommendations 2023 (NNR2023) project (6)
- The papers are included in the extended NNR2023 report but, for transparency, these scoping reviews are also published in Food & Nutrition Research
- The scoping reviews have been peer reviewed by independent experts in the research field according to the standard procedures of the journal
- The scoping reviews have also been subjected to public consultations (see report to be published by the NNR2023 project)
- The NNR2023 committee has served as the editorial board
- While these papers are a main fundament, the NNR2023 committee has the sole responsibility for setting dietary reference values in the NNR2023 project

## Methods

The review follows the protocol developed within the NNR2023 project (6). Initially, a literature search was conducted using the MeSH term Manganese, focusing on reviews in human subjects published from January 2011 to February 2022. The search resulted in 221 records out of which 27 were included for review. In addition to the review articles retrieved from PubMed, several expert opinion documents were included as sources of evidence (1, 3–5, 7, 8). Lastly, key references found within identified review articles, expert opinion documents, or identified for specific purposes have also been cited herein.

## Physiology

In adults, it has been estimated that the average gastrointestinal absorption of manganese is below 10% (4, 9). The intestinal absorption has been suggested to occur both via active transport and passive diffusion (4) although incompletely characterized (10). The amount of manganese absorbed in the gastrointestinal tract appears to be influenced by the concentration in the diet (11, 12), suggesting that the regulation at the absorption level is part of the adaptive response that maintains manganese homeostasis over a wide range of intakes (4). In addition, the gastrointestinal absorption of manganese has been shown to differ by sex (13), with women absorbing significantly more manganese from the diet than men. This may be related to that iron deficiency with concomitant upregulated iron uptake is more common in women than in men, and that iron and manganese may utilize the same transporters during iron deficiency, for example, the divalent metal transporter-1 (DMT-1) (10). In addition, epidemiological studies have reported an inverse association between serum ferritin and the manganese concentration in blood (11–-14). However, under normal circumstances the two metals appear to be handled differently, transported by different transporters, and regulated by independent homeostatic signals (10). Under experimental conditions, the gastrointestinal absorption of manganese has been shown to increase by the presence of zinc, and decrease in relation to dietary fibre, phytate, copper, and calcium (15). The absorption of manganese is also dependent of age, with infants and young children absorbing more from the diet than adults, and the retention in the body is also longer (9, 15).

Once manganese enters the portal circulation in the form of Mn^2+^ (more than 99%), it primarily binds to alpha2-macroglobulin or albumin, while the minor fraction of manganese in the form of Mn^3+^ is bound to transferrin (3). In the bloodstream, more than 60% of the manganese is found in erythrocytes, about 30% in leukocytes and platelets, and only around 4% in plasma (16). The manganese concentration in blood in healthy adults has been reported to vary between 4 and 15 µg/L (1). Higher blood concentrations have been reported among pregnant women (range of means 15–20 µg/L), and cord blood concentrations have been reported to be about 2- to 3-fold higher than those of maternal blood (4).

No specific storage organs or storage forms have been identified for manganese (4). Commonly, the highest concentrations of manganese are found in the liver, kidney, and pancreas. In the cells, manganese is mainly found in the mitochondria and nuclear fractions. Manganese uptake into a specific cell type appears to be dependent on the oxidation state of manganese (Mn^2+^ or Mn^3+^) and on the specific transport systems expressed in that cell type. The brain has been identified as the critical target organ of manganese toxicity. Following oral exposure, manganese can enter the brain either through the blood-brain-barrier (BBB) or via the blood-cerebrospinal fluid (CSF) barrier. Manganese appears to be transported into the brain via several different importers, including DMT-1, transferrin and the transferrin receptor, calcium channels, citrate transporters, and metal cation symporter ZIP8 (encoded by *SLC39A8*) and metal cation symporter ZIP14 (*SLC39A14*) (17). Compared to cellular uptake, less is known about the cellular export of manganese. Zinc transporter 10, ZnT-10 (*SLC30A10*), is one of the few identified Mn^2+^ efflux transporters, and it is expressed in the liver, in enterocytes, and in certain parts of the brain. Zinc transporter 10 and metal cation symporter ZIP8 are assumed to be critical for the regulation of manganese as loss of function mutations in the *SLC39A8* or *SLC30A10* genes are associated with severely dysregulated manganese homeostasis and neurological symptoms (3). Also, common genetic variations in these genes have been suggested to influence the concentrations of manganese and manganese toxicity in different life stages (18, 19).

The half-life of manganese in the body has been shown to vary between 13 and 37 days (3). It has been shown to be longer in men than in women (13), which as for the intestinal absorption has been suggested to be related to sex differences in iron status. In addition, a higher dietary intake was associated with a shorter half-life (12), suggesting that the elimination is part of the homeostatic response to varying dietary intake. Manganese is primarily eliminated via the bile into the intestine followed by excretion via faeces (4, 9). Very little manganese is excreted in urine (about 1% of the dietary intake), and concentrations usually range between 1 and 8 µg/L (1).

Based on data from 1990 to 2012, the mean manganese concentration in breast milk has been reported to vary between 0.8 and 30 µg/L (4). In another extensive review, based on data from 1980 to 2017 resulting in 5,423 breast milk samples, the mean concentration was 7.7 µg/L (range: 0.17–30.27 µg/L) (20). The concentration is higher in colostrum than in mature milk. The concentration in mature milk has been reported to be rather stable up until 6 months post-partum (4). Several studies have reported that there is no correlation between maternal dietary intake of manganese and the concentrations in breast milk (4).

## Assessment of nutrient status

Several biomarkers have been used in attempts to develop accurate indicators that are able to reflect total exposure to manganese, for example, blood, faeces, urine, hair, nails, teeth, and bone as well as manganese-dependent enzymes.

Manganese concentrations in blood (e.g. whole blood, plasma, or serum) have, on a group level, been shown to respond to large variations in recent dietary intake (15). On the other hand, in a study comparing individuals who were not consuming tea (mean dietary intake 3.2 mg/day) with tea drinkers (mean intake 5.5 mg/day or 10 mg/day depending upon assumed manganese concentration of black tea), no significant differences were observed in either plasma or whole blood manganese concentrations between the two groups (21). Thus, in combination with studies showing that manganese in blood is dependent on polymorphisms in genes (for example *SLC30A10*) encoding manganese transporters (19), it may be questioned whether manganese in blood can reflect small differences in dietary intake.

As manganese is mainly excreted in faeces and only a small amount of the dietary intake is absorbed in the gastrointestinal tract, faeces has been proposed as a marker of recent dietary intake (4). Nevertheless, the practical use is limited as faecal manganese represents the sum of both unabsorbed dietary manganese and the amount excreted via bile and is thereby influenced by several factors (e.g. dietary composition, previous intake). Manganese in urine is rarely used in studies evaluating potential health effects, most likely because only a small fraction is excreted in urine and the variability in urine is high (3). There are a few studies that have reported an association between dietary intake and concentrations in urine, while other studies found no association (15).

Manganese concentrations in hair and nails have been used as markers of more long-term exposure. Some studies have found an association between the concentration in hair and dietary intake, while others have not and there is no correlation with concentrations in blood (15). The latter is likely due to the fact that hair concentrations reflect long-term exposure, whereas blood reflects recent exposure. The major limitation with hair is the risk of external contamination, and despite advanced cleaning methodologies (22), it cannot be excluded that external manganese can bind irreversibly to hair. Toenail concentrations, which might be less prone to external contamination, seem to represent long-term total exposure with fairly good reproducibility, but the limited data do not suggest any association with dietary intake (23).

As manganese accumulates in bone, it may also be a biomarker of long-term exposure although available data is only based on occupational exposure (3). Concentrations in deciduous teeth can provide information concerning the timing and intensity of exposure during pregnancy (foremost second and third trimester), early childhood, and early life (3). However, like in blood, the levels in teeth have been shown to be influenced by polymorphisms in genes (*SLC39A8* and *SLC30A10*) encoding manganese transporters (18).

Manganese-dependent enzymes, in particular MnSOD, have been explored as potential biomarkers. In humans, high manganese intakes have been associated with increased MnSOD activity, but as the activity is also influenced by other factors it lacks specificity (4).

In summary, there are no reliable and validated biomarkers of manganese intake or status.

## Dietary intake in Nordic and Baltic countries

There are several dietary sources that have been shown to be rich in manganese, including nuts, chocolate, cereal-based products, shellfish, pulses, and fruit and fruit products (4). Data from France and the UK (4, 24, 25) identified that the main contributors of manganese were cereal-based products, vegetables, fruit and fruit products, and beverages (coffee, tea, alcoholic beverages). Similarly, in the Swedish market basket study in 2015, the main contributor was cereals (57%) (26). Drinking water may also contain elevated concentrations of manganese (5). The recent EU drinking water directive has set a parametric value of 50 µg/L for manganese as an indicator parameter, which has no direct public-health impact but it is important for the functioning of water facilities and water quality (27). In the Nordic and Baltic countries, drinking water from public waterworks normally contains a manganese concentration below 50 µg/L. However, in Denmark, episodic drinking water treatment failures have sometimes been shown to result in manganese concentrations above 100 µg/L (28). In Sweden, highly varying manganese concentrations have been reported in drinking water from private wells (641 samples collected nationwide), with 2.5% even exceeding 400 µg/L (29). Elevated manganese concentrations in drinking water have also been reported in certain areas in Norway, Finland, and Estonia (30–32), while in Iceland concentrations above 50 µg/L have not been reported (33).

Balance studies have reported maintained manganese balance at intakes below 2.5 mg/day and null or positive balance at intakes above 2.5 mg/kg in 11–60 days studies, suggesting that the body seems to adapt rather quickly to large variations in manganese intake (4). However, it has also been noted that comparison between individuals and studies may be difficult as the manganese balance seems to be influenced by the type of diet, adaptation to varying dietary intake, and individual differences in rate of absorption or excretion and body content (4).

There are a few published studies on the dietary intake of manganese in the Nordic countries. The mean manganese intake of Finnish children and adolescents, aged 3–18 years, was in the range of 3–7 mg/day calculated from food consumption data (48-h recall) and food contents (34). In the Swedish market basket study in 2015 (26), the daily estimated per capita intake of manganese was 4.2 mg per person, which is very similar to the previous market basket study in 2010 with a corresponding intake of 4.0 mg (35). In a Danish study of 100 men who collected duplicate portions of their regular diets for 48 h, the median dietary intake of manganese was 3.9 mg/day (36). In a national Danish dietary survey in 2011–2013, including individuals aged 4–75 years and assessing dietary intake through a 4-day food record, the median (95-percentile) intake of manganese was 2.1 mg/day (4.3) in 1–3 years old children and 3.9 mg/day (6.9) in women aged 65–75 years of age (8). In summary, the manganese intakes are within the range of intakes of adults in the EU, ranging from 2 to 6 mg/day (4).

In a Finnish study of exclusively breast-feed infants the median estimated daily intake of manganese at 1, 2 and 3 months of age was 0.9, 0.6 and 0.5 µg/kg body weight per day (corresponding to 3–4 µg/day) (37). In a Swedish study, the mean breast milk concentration of manganese at 2–3-week post-partum was 3 µg/L (38), which is in the lower range of that reported from seven other studies in Europe where mean concentrations range between 3 and 30 µg/L (4). Based on this information, and assuming an average milk intake of 0.8 L/day, the mean intake of exclusively breastfeed infants up to 6 months of age would range between 2.4 and 24 µg/day (4). In a recent review (20), the daily intake of manganese of infants and young children fed infant formula, sampled in market basket surveys of products in France and the US, was estimated assuming no contribution of manganese from water. In France, the minimum and maximum intakes from formula were 150 and 340 µg/day at 3 weeks of age, 230 and 550 µg/day at 4.25 months, 140 and 370 µg/day at 7 months, and 68 and 190 µg/day at 18 months of age. Likewise, large variations in daily intake of manganese from different types of infant formulas (minimum-maximum: 15–2,256 µg/day) have previously been reported in Sweden (39, 40).

Many multivitamin-mineral and mineral supplements for adults contain manganese. The Swedish Food Agency has reported that most supplements on the Swedish market contain between 0.5 and 4 mg per daily dose (41).

## Health outcomes relevant for Nordic and Baltic countries

In general, there is limited information concerning the relationship between manganese intake or status and health-related endpoints or disease prevention. Also, none of the identified studies have been conducted in the Nordic or Baltic countries.

### Deficiency

In various animal species, manganese deficiency has been associated with impaired growth, skeletal abnormalities, reproductive deficits, impaired glucose tolerance, and alterations in carbohydrate and lipid metabolism (7). Manganese deficiency in humans has only been documented under experimental settings (42). In a manganese depletion-repletion study, seven young men, aged 19–22 years, were fed a conventional manganese diet of 2.59 mg/day for 3 weeks to establish a baseline followed by a purified diet of 0.11 mg/day of manganese for 39 days (depletion) and thereafter 5 days periods of 1.53 and 2.55 mg/day of manganese (repletion) (42). At the end of the depletion period, five out of the seven males developed fleeting dermatitis and miliaria crystallina, which disappeared during repletion. Also, plasma cholesterol levels declined during baseline and the depletion period.

### Neurological outcomes after elevated intake

In the comprehensive literature review conducted by the European Food Safety Authority (EFSA) prior to their evaluation in 2013 (4, 43), including studies between 1990 and 2011, a small cross-over study was identified in which healthy young women (21–46 years of age) had consumed diets containing a low or high manganese concentration (0.8 mg versus 20 mg per day) combined with two sources of fat for 8 weeks, with no changes in neurological or other physiological outcomes (12). Besides this study, no other studies have been identified which have assessed associations of the dietary intake of manganese and neurological outcomes in either adults or children. However, there are several epidemiological studies that have explored the link between manganese exposure from all sources, assessed through suggested manganese biomarkers (hair, nails, blood and teeth), and neurodevelopmental outcomes in children and teenagers (44, 45). Nevertheless, most of the studies have a cross-sectional design and they have been conducted in areas with elevated manganese concentrations in groundwater or air emissions. In a systematic review and meta-analysis, including studies up to the end of 2019 (45), results from cohort studies, assessing mainly prenatal manganese biomarkers (e.g. maternal blood, cord blood, and teeth), reported predominantly negative associations with cognitive and motor skills in children below 6 years of age (45). Cross-sectional studies indicated negative associations between manganese concentrations in hair (most often a mean concentration above 0.55 µg/g), but not in blood or teeth, and cognitive and behavioural outcomes in children aged 6–18 years. In summary, the limited number of prospective studies, and the lack of well-established biomarkers of manganese exposure and common validated neurodevelopmental outcomes render data uncertain and inconclusive.

### Other health endpoints

As previously mentioned, manganese is a cofactor for enzymes involved in bone formation as well as glucose, carbohydrate, and lipid metabolism. There are a few studies that have reported that women with osteoporosis have lower manganese concentrations in serum compared to women with normal bone density (46). Also, some studies have suggested non-linear relationships of urinary and blood manganese concentrations with metabolic syndrome, and that higher dietary intake of manganese may be associated with a lower prevalence of metabolic syndrome (47). Likewise, a U-shaped association has been reported between plasma manganese concentrations and type 2 diabetes (48). Nevertheless, most of the studies are methodologically limited by the cross-sectional design and potential for confounding.

## Requirement and recommended intakes

In 2001, the Institute of Medicine (IOM; named National Academy of Sciences, Engineering, and Medicine since 2015) concluded that there was insufficient data to set an estimated average requirement (EAR) as a wide range of manganese intakes can result in manganese balance (7). Also, as manganese deficiency was not apparent in the North American population, a recommended dietary allowance (RDA) based on balance data would overestimate the requirement. Instead, adequate intakes (AI) were established for different age groups (Table 1), primarily based on median intakes reported from the U.S. Total Diet Study in 1991–97. From the age of 9–13 years, males have marginally higher manganese intake than females. In adults, the recommended AI is 2.3 mg/day for males and 1.8 mg/day for females. The AI during pregnancy was set to 2.0 mg/day based on extrapolation considering a weight gain of 16 kg, and for lactating women 2.1 mg/day is based on the median intake during lactation. The AI of infants aged 0–6 months is based on the average concentration of manganese in breast milk (3.5 µg/L) and an average milk consumption (0.78 L/day), resulting in 3 µg/day. The AI of older infants was set to 0.6 mg/day based on estimated consumption of infants in combination with extrapolation from adults.

**Table 1:** Adequate intakes for manganese from IOM (7)

Age	Adequate intake males (mg/day)	Adequate intake females (mg/day)
0–6 months	0.003	0.003
7–12 months	0.6	0.6
1–3 years	1.2	1.2
4–8 years	1.5	1.5
9–13 years	1.9	1.6
14–18 years	2.2	1.6
>18 years	2.3	1.8
Pregnancy		2.0
Lactation		2.1

In 2013, EFSA concluded that data was insufficient for deriving average requirements or population reference intakes (4). This was based on that there were no suitable biomarkers of manganese status, that balance can be achieved across a range of intakes, and that there were no studies on intake or status in relation to health endpoints that could be used to derive dietary reference values. Thus, in line with IOM, an AI for adults was set at 3.0 mg/day based on mean intakes of manganese from mixed diets within the EU (Table 2). In contrast to IOM, it was considered unnecessary to provide sex-specific values. It was suggested that the AI for adults should also apply for pregnant and lactating women. The AIs for children and adolescents were extrapolated from the adult values using isometric scaling and the average body weight for each age group. For infants, the lower value of 0.02 mg/day is based on the intake of breast-fed infants (mean concentration of 15 µg/L and a consumption of 0.8 L/day) and extrapolation using isometric scaling, while the higher value of 0.5 mg/day is a combination of intake among infants aged 6–12 months using a reference body weight and extrapolation of adult AI by isometric scaling.

**Table 2:** Adequate intakes of manganese from EFSA (4)

Age	Adequate intake (mg/day)
7–11 months	0.02–0.5
1–3 years	0.5
4–6 years	1.0
7–10 years	1.5
11–14 years	2.0
15–17 years	3.0
≥18 years	3.0

The NNR2012 did not include recommendations for manganese intake (49). Herein, the evidence regarding dietary intake of manganese and health-related endpoints was considered too limited and inconclusive. Thus, requirements are still difficult to determine.

Future research should foremost focus on identifying a reliable biomarker of manganese status. There is also a need of large prospective studies assessing manganese intake in susceptible groups, such as bottle feed infants, and the association with neurodevelopment which has been proposed to be the most critical endpoint with regard to excessive intake. There is also a need for more prospective studies exploring the role of manganese intake in relation to the risk of developing metabolic syndrome (47), diabetes (48), and osteoporosis (46).
